# Influence of blood coagulability after spinal surgeries

**DOI:** 10.1590/1413-78522014220500930

**Published:** 2014

**Authors:** Marcelo Hide Matsumoto, Luiz Claudio Lacerda Rodrigues, Luiz Gustavo da silva Batalini, Thales Arcanjo Fonteles, Adalberto Bortoletto

**Affiliations:** 1 Hospital Santa Marcelina, Department of Orthopedics and Traumatology, São Paulo, SP, Brazil, Department of Orthopedics and Traumatology, Hospital Santa Marcelina, São Paulo, SP, Brazil

**Keywords:** Orthopedics, Spine, Blood coagulation disorders, Thrombosis

## Abstract

**Objective:**

To verify whether spinal surgery causes relevant changes in the blood clotting process and define which factors have the greatest influence on changes found.

**Method:**

This is a not randomized, cross-sectional study, Forty seven patients were evaluated between August 2011 and February 2013, whose clinical, surgical, laboratory and image daata were collected. The data obtained were crossed with the epidemiological data of each patient in a moment prior to and another after surgery searching which variables have been directly influenced.

**Result:**

Our analysis showed that the most important changes occurred in patients with BMI classified, according to the World Health Organization (WHO) as out of healthy range. Other smaller correlations were also found. Another important consideration was the tendency to observe hypercoagulability in smoker patients, a fact that is not influenced by spinal procedures.

**Conclusion:**

We concluded that spinal surgeries cause few relevant changes in the blood clotting process and that among the factors studied, BMI (when out of the healthy range, according to the WHO classification) showed closer relationship with changes in laboratory coagulation tests. *Level of Evidence III, Cross-Sectional Study. *

## INTRODUCTION

Thromboembolism, including deep vein thrombosis and pulmonary embolism, was initially described by Rudolph Virchow in 1850 apud Heck *et a*l,^1^ the triad which is known as Virchow's Triad.; it consists of hypercoagulability, venous stasis and to the venous intimal injury.^2^ Today we know that other situations may compromise coagulation as mutation of Leiden Factor V, C, S and antithrombine III factors deficiency, among others; besides acquired conditions such as hormone replacement, late pregnancy and tumors.^1^

In spine surgery, the factors for venous stasis are: long time horizontal ventral decubitus, lack of muscle tone, venous compression by retractors and postoperative bed rest. Venous intimal injury may occur in surgical handling.^1^

Coagulation problems, so well-studied in cases of hip and knee arthroplasties, previously considered rare in spinal surgery, may not be that rare.^3^^,^ 4 The literature refer both to cases of excessive bleeding as deep vein thrombosis and pulmonary embolism, the latter being reported in greater number.^5^

Due to the small number of studies on the subject, we question whether there is a true relationship between surgical manipulation of the spine and coagulatory changes described. A precise answer is difficult due to the numerous variables, from patient-related factors such as gender, age, ethnicity and lifestyle habits; going by surgical, traumatic or atraumatic variables, access routes, surgical time and manipulated levels; until apparently detailed environmental variables such as room temperature in the peri-operative period, anesthetic recovery time and training of support staff. Therefore, it has not yet been possible to properly establish which are the risk factors for excessive bleeding or thrombosis in patients undergoing these procedures, being available only some evidences.^6^^-^ 8

The diagnosis of these complications can be difficult, many patients remain asymptomatic despite having undergone considerable bleeding or showing vein thrombosis confirmed by Doppler of lower limbs - so there are variances in the studies on which criteria should be used to confirm the actual existence of the surveyed changes; simple tests such as dosage of hemoglobin (Hb) and hematocrit (Ht), platelet count, coagulogram, fibrinogen and D-dimer dosage are among the most used, some require more complex tests such as duplex scan of the lower limbs, angiography, venography and thoracic CT.^5^^,^ 9 ^-^ 11

Studies with other types of orthopedic surgery indicate that the incidence of thromboembolic complications can reach 70% of pacientes.^12^ The incidence of symptomatic patients is around 0.5 to 2.5% of the procedures in the spine, but it is estimated the incidence of asymptomatic patients to exceed 15%.^13^

This work aims to identify whether spinal surgery can cause significant changes in the blood clotting process and, thereafter, determine which factors have the greatest influence on changes found.

## MATERIALS AND METHODS

This is a cross-sectional study, which evaluated nonrandomized patients undergoing surgical treatment of spinal diseases.

There are no conflicts of interest in this study and it was approved by the local Research Ethics Committee.

Inclusion criteria were: all patients undergoing spinal surgery at our institution between August 2011 and February 2013, aged 18 years or more at surgery, of both genders, who accepted the terms set out in the study and signed the Free and Informed Consent Form (FICF). Exclusion criteria were patients who had no cognition to inform the data, with previous history of thromboembolic complications, which used anticoagulant medication continuously over the last six months before surgery, who had peripheral vascular disease with ulcers or who had diseases of the hematopoietic system.

From each patient a complete hemogram, coagulogram and fibrinogen count was collected prior to surgery (preoperative) and on the 2^nd^ day postoperatively. During hospitalization, patients were evaluated every day by the same physician, in search of clinical changes consistent with thromboembolic events: swelling of the calves, lower limb edema, and dyspnea without any other cause. After hospital discharge, each patient underwent a Doppler ultrasonography of the lower limbs between the seventh and fourteenth day postoperatively.

The data were analyzed together with the epidemiological data of each patient - age, gender, ethnicity, body mass index (BMI), previous history of treatment for cancer, surgery for traumatic or atraumatic causes, prior hormone replacement therapy, site of surgery, access route, number of manipulated levels, tobacco use, duration of surgery, estimated bleeding during the procedure and number of packed red blood cells administered - in order to obtain its statistical significance.

After preliminary statistical analysis, the variables of interest were defined and excluded those that were not significant enough to prove direct influence of the surgical procedure. Thus, the variables of interest were taken for inferential analysis in the following manner based on the likelihood ratio tests.

BMI (According to the WHO classification),^14^ less than 18.5 is considered underweight; between 18.5 and 24.9, healthy; between 25 and 29.9, overweight; and greater than or equal to 30, obese.

Surgery time: being less than 180 minutes, or greater than or equal to 180 minutes;

Change in hemoglobin after the procedure: considered as less than 3.4g/dL or greater than or equal to 3.4g/dl.

For each case, we set a model of simple linear regression,^15^ considering the difference between post and pre-operative value of the variables of interest.

The variables of interest were, then, analyzed by performing the intersection of the data all together, being directly excluded those who had value greater than 5% in the likelihood ratio test, after that, the remaining variables were analyzed together and individually for their statistical significance, considered sufficient when p <0.001.

## RESULTS

The sample comprised 29 men and 18 women, a correlation of 1.6:1. Regarding ethnicity, 25 patients (53.2%) considered themselves white, 13 (27.7%) mixed, and nine (19.1%) reported themselves as black.

The mean age of patients was 46 years old (range 18 to 74 years old) and the median was 50 years old. Of these, 27 allegedly never smoked (57.4%) and 20 were smokers or ex-smokers (42.6%).

Of the operated patients, 44 had never been treated for cancer (93.7%), two had been treated for breast cancer (4.3%) and one for cervical cancer (2%).

Of the 18 women, four (22.2%) were performing or have had some type of hormone replacement therapy (HRT).

As for BMI, 23 patients (48.9%) were classified as overweight, 14 (29.8%) within the healthy range, eight (17%) obese and two (4.3%) were considered underweight.

The most operated anatomical region was the lumbosacral transition with 16 procedures (34%) followed by the thoracolumbar transition with 14 (29.8%), lumbar with nine (19.1%), cervical, six (12.8%) procedures and chest, two surgeries (4.3%). No patient underwent the procedure in the cervicothoracic transition.

Seventeen surgeries (36.2%) were performed by traumatic causes, and 30 (63.8%) by non-traumatic causes.

One level was manipulated in three patients (6.4%), two levels in 18 patients (38.3%), three levels in 11 patients (23.4%), four levels in 13 patients (27.7%) and only two cases were handled more than five levels (4.2%).

Of the forty seven surgeries, three were performed by anterior access route (6.4%) and 44 by posterior access route (93.6%). The surgical procedure, from the time of placing the anesthetized patient on the surgical table to removal, had duration shorter than 180 minutes in 19 patients (40.4%) and greater than or equal to 180 minutes in 28 patients (59.6%).

There was a drop in Hb levels lower than 3.4 points in 22 patients (46.8%) and greater than or equal to this value in 25 patients (53.2%). During surgery and/or on the 1^st^ day after surgery, 12 patients (25.6%) received blood transfusion and in 35 patients (74.4%) there was no such need.

All patients included presented themselves before the procedure with full peripheral pulses and no edema of the lower limbs. When evaluated at 1 and 2 days after the procedure, six patients (12.8%) had edema of the lower limbs, and seven (14.9%) presented decreased peripheral pulses. None showed swelling of the calves after surgery.

Fibrinogen levels ranged, on average, 232 mg/dL before surgery to 406 mg/dL after it. The decrease in mean platelet count was 215,000 cells/mm³ before surgery to 192,000 cells/mm³ after surgery. We also acknowledge the average decrease in prothrombin activity (PA) of 93.4% to 83.6%, the average international normalized ratio (INR) ranged from 1.02 to 1.09 prior to surgery and after the ratio of APTT ranged on average from 0.92 to 0.9 before and after the procedure, respectively.

Venous Doppler of the lower limbs showed only one patient (2.12%) with thrombosis.

The variables used are exemplified in Table1. After statistical analysis, for each variable, we obtained an interpretation:

Fibrinogen - we observed that the influence of BMI of the patients operated on the change in value of fibrinogen does not have sufficient statistical power (p = 0.216), as well as the influence of time of surgery (p = 0.599) and total number of operated levels (p = 0.821).

**Figure 1:**
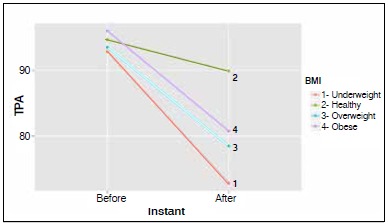
Change of PA according to patients&apos; BMI.

PA (prothrombin activity) - we noticed that the influence of procedure time, number of operated levels and decreases in Hb did not affect the variation of this laboratory test significantly (p = 0.63, 0.34 and 0.08, respectively). When we observe the influence of BMI on this examination, we found that patients classified as healthy regarding BMI showed no significant difference between pre and post-surgery (p = 0.1). Patients with BMI classified as underweight, tended to a higher variation in postoperative PA, however, with p slightly bigger than 0,001. Patients already classified as overweight and obese had clear influences on PA variation (p <0.001). Figure 1 shows the decrease in postoperative values of PA, more pronounced in patients with a BMI outside the healthy range.

**Figure 2:**
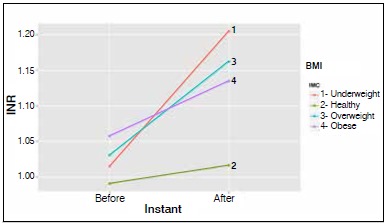
Confrontation between BMI and INR variation.

INR - this test was not influenced by the decrease in Hb levels

(p = 0.069), the estimated intraoperative bleeding (p = 0.889), or the changes seen on physical examination (p> 0.001). However, BMI levels showed once again to relate with INR. Patients with a BMI classified as healthy showed no significant difference in the influence of the variation of INR pre and post-surgery (p>0.001). Patients with BMI underweight and obese had a greater tendency toward variation of INR, but with p slightly over 0,001. Those with overweight BMI were directly related to changes in INR (p<0.001). Figure 2 shows the changes in the value of postoperative INR, more pronounced in patients with a BMI outside the healthy range.

Ratio - The activated partial thromboplastin time (APTT) evaluates the activation of the intrinsic coagulation pathway through a daily laboratory control obtained through the ratio APTT (patient)/ APTT (control). All correlations within a 5% level were not sufficient to obtain p <0.001.

**Table 1:** Values of variables studied pre- and post-surgery

Variable	Minimum	Median	Maximum	Mean
Age (years old)	18	50	74	46.77
Weight (Kg)	37	76	114	75.21
BMI	14.45	26.84	37.80	26.54
Time of Surgery (min)	120	180	300	183.62
Ht (pre)	30.3	40.1	49.8	40.13
Hb (pre)	10.5	13.7	17.2	13.67
Fibrinogen (pre)	196	232	425	244.02
TPA (pre)	63.8	93.4	130.9	94.30
Ratio (pre)	0.72	0.92	1.32	0.95
Platelets (pre)	116.000	215.000	463.000	236.617
INR (pre)	0.8	1.02	1.32	1.02
Ht (post)	20.4	30	37.1	28.97
Hb (post)	6.9	10.1	12.9	9.98
Fibrinogen (post)	218	406	688	411.11
TPA (post)	56.2	83.6	103	82.04
Ratio (post)	0.74	0.9	1.23	0.93
Platelets (post)	94.000	192.000	434.000	205.872
INR (post)	0.94	1.09	1.44	1.12

**Figure 3:**
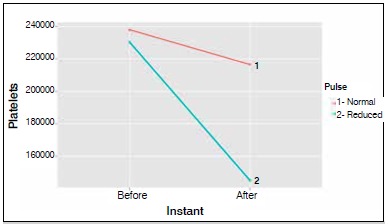
Relationship between fall of platelets level and change in peripheral pulse.

Platelets - the analysis of the fall in platelet count with the other variables indicated a link with decreased peripheral pulses after surgery (p <0.001), the other variables were not statistically significant (p> 0.001), as shown in Figure 3.

**Figure 4:**
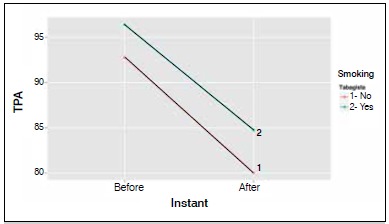
Relationship between variation of prothrombin activity in smoking and non-smoking patients.

**Figure 5:**
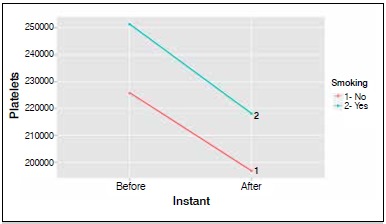
Relationship between fall of platelets level and among smoking and non-smoking patients.

**Figure 6:**
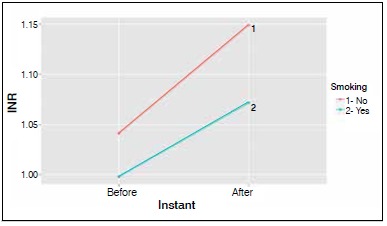
Relationship between variation of INR among smoking and non-smoking patients.

**Figure 7:**
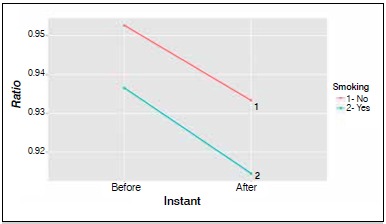
Relationship between variation of Ratio (APTT) among smoking and non-smoking patients.

Smoking - We observed that smokers show the percentage of activation of prothrombin (PA) and platelet count values greater than nonsmokers, both before and after surgery. Likewise, smokers show lower INR and ratio than non- smokers.

(Figures 4, 5, 6 and 7)

## DISCUSSION

In our study the analysis of gender and mean age was consistent with that found in other publications.^3^^,^ 5 Regarding the increased surgical time, we cannot establish a direct correlation with increased incidence of coagulatory changes, as some authors claim.^3^

The anatomical portion most operated in our work was the lumbosacral spine transition (34%), followed by the thoracolumbar transition (29.8%), confirming that these are the regions of greatest vulnerability of the spine.^5^

The comparative study of data collected before and after the surgical procedure compared to other variables lead us to establish which changes are influenced by surgery. We, thus, observed that the relationship between BMI and coagulation studies were those who had a higher level of significance (p <0.001). Overweight and obese patients were those with the most important coagulatory changes, all tending to an exaggerated activation of the coagulation cascade (falling values of AP and increased INR by consumption of coagulation factors) and possible tendency to thromboembolic complications. For underweight patients, the curve does not change with such intensity, but retains a similar pattern. Patients considered in a healthy BMI range did not show any significant laboratory abnormalities.^14^

Among the 47 patients, 42.6% were smokers, a data which might be a little below the rates found in our references.^5^ It drew our attention the laboratory tests of smokers to be always present in a hypercoagulable state compared to non-smokers (increased values of platelet count and PA, and decreased INR and ratio) seen before and after surgery, which does not appear to be directly influenced by it.

A relationship observed with a good statistical significant was the decrease in the peripheral pulses in patients who had sharp fall in the platelet count after surgery.

The other variables followed an expected pattern already confirmed by the physiology of human clotting, so, there is no need for prophylaxis of thromboembolic events in postoperative in previously healthy, non-obese, nonsmoker patients.

In our series only one patient had a thromboembolic event confirmed by Doppler ultrasound examination of the lower limbs. This finding is consistent with most of the references cited, but there are reports of higher indexes.^8^^,^ 16 ^-^ 18 We, thus, can define that this test is not suitable for use as tracking of thromboembolic events due to its cost and availability, which should be used only in patients with compatible

clinical findings.

We also observed that pre-established situations on coagulation physiology were confirmed, such as variations in hematocrit and postoperative hemoglobin compatible with the amount of blood loss and the time of the procedure, in addition to activation of the coagulation cascade and consumption of factors leading to changes observed within the normal range of laboratory tests.^2^

## CONCLUSION

We conclude that spine surgeries cause a few significant changes in the blood clotting process and that among the factors studied, BMI, when outside the healthy range by the WHO classification showed a greater relationship with the change in laboratory coagulation tests. We also observed that smokers have their clotting factors changed previously to surgical intervention.
